# Does the Identification of a Minimum Number of Cases Correlate With Better Adherence to International Guidelines Regarding the Treatment of Penile Cancer? Survey Results of the European PROspective Penile Cancer Study (E-PROPS)

**DOI:** 10.3389/fonc.2021.759362

**Published:** 2021-11-29

**Authors:** Steffen Lebentrau, Gamal Anton Wakileh, Martin Schostak, Hans-Peter Schmid, Rodrigo Suarez-Ibarrola, Axel S. Merseburger, Georg C. Hutterer, Ulrike H. Necknig, Michael Rink, Martin Bögemann, Luis Alex Kluth, Armin Pycha, Maximilian Burger, Sabine D. Brookman-May, Johannes Bründl, Matthias May

**Affiliations:** ^1^ Department of Urology, Werner Forßmann Hospital, Eberswalde, Germany; ^2^ Department of Urology, Ulm University Hospital, Ulm, Germany; ^3^ Department of Urology and Urooncology, University Medical Center Magdeburg, Magdeburg, Germany; ^4^ Department of Urology, School of Medicine, University of St. Gallen, St. Gallen, Switzerland; ^5^ Department of Urology, Faculty of Medicine, University of Freiburg Medical Centre, Freiburg, Germany; ^6^ Department of Urology, University of Schleswig-Holstein, Lübeck, Germany; ^7^ Department of Urology, Medical University of Graz, Graz, Austria; ^8^ Department of Urology and Pediatric Urology, Klinikum Garmisch-Partenkirchen, Garmisch-Partenkirchen, Germany; ^9^ Department of Urology, University Medical Center Hamburg-Eppendorf, Hamburg, Germany; ^10^ Department of Urology and Pediatric Urology, University Medical Center Münster, Münster, Germany; ^11^ Department of Urology, University Medical Center Frankfurt a.M., Frankfurt/Main, Germany; ^12^ Department of Urology, Hospital of Bolzano, Bolzano-Bozen, Italy; ^13^ Medical School, Sigmund Freud University Vienna, Vienna, Austria; ^14^ Department of Urology, Caritas St. Josef Medical Centre, University of Regensburg, Regensburg, Germany; ^15^ Department of Urology, University Hospital Großhadern, Ludwig-Maximilians-University Munich, Munich, Germany; ^16^ Department of Urology, St. Elisabeth Hospital Straubing, Brothers of Mercy Hospital, Straubing, Germany

**Keywords:** penile neoplasms, guideline adherence, organ-sparing treatment, lymph node dissection, chemotherapy

## Abstract

**Background:**

Penile cancer represents a rare malignant disease, whereby a small caseload is associated with the risk of inadequate treatment expertise. Thus, we hypothesized that strict guideline adherence might be considered a potential surrogate for treatment quality. This study investigated the influence of the annual hospital caseload on guideline adherence regarding treatment recommendations for penile cancer.

**Methods:**

In a 2018 survey study, 681 urologists from 45 hospitals in four European countries were queried about six hypothetical case scenarios (CS): local treatment of the primary tumor pTis (CS1) and pT1b (CS2); lymph node surgery inguinal (CS3) and pelvic (CS4); and chemotherapy neoadjuvant (CS5) and adjuvant (CS6). Only the responses from 206 head and senior physicians, as decision makers, were evaluated. The answers were assessed based on the applicable European Association of Urology (EAU) guidelines regarding their correctness. The real hospital caseload was analyzed based on multivariate logistic regression models regarding its effect on guideline adherence.

**Results:**

The median annual hospital caseload was 6 (interquartile range (IQR) 3–9). Recommendations for CS1–6 were correct in 79%, 66%, 39%, 27%, 28%, and 28%, respectively. The probability of a guideline-adherent recommendation increased with each patient treated per year in a clinic for CS1, CS2, CS3, and CS6 by 16%, 7.8%, 7.2%, and 9.5%, respectively (each *p* < 0.05); CS4 and CS5 were not influenced by caseload. A caseload threshold with a higher guideline adherence for all endpoints could not be perceived. The type of hospital care (academic vs. non-academic) did not affect guideline adherence in any scenario.

**Conclusions:**

Guideline adherence for most treatment recommendations increases with growing annual penile cancer caseload. Thus, the results of our study call for a stronger centralization of diagnosis and treatment strategies regarding penile cancer.

## Introduction

In Europe, the age-standardized incidence rate for penile cancer in 2018 was 0.9/100,000 (1). Penile cancer represents a rare malignant disease, whereby due to a low annual caseload, most centers have limited experience in the accurate management of such patients (2). Hence, it is difficult to conduct prospective randomized studies on penile cancer since the evidence supporting guideline recommendations and the research interest are scarce (3). However, an evaluation of guideline recommendations may help pool the currently reported expertise.

Guideline adherence in the treatment of penile cancer is low (4, 5). In an evaluation of the Swedish National penile cancer register from 2000 to 2012, Kirrander et al. found 71% guideline adherence for organ-preserving surgery and 50% for lymph node dissection (4). Moreover, according to the *Surveillance, Epidemiology, and End Results Program* from 1998 to 2015, guideline adherence for inguinal lymphadenectomy (ILND) did not reach 25% (5). Concurrently, several studies support the association between annual caseload and outcomes, suggesting that improved guideline adherence has a beneficial impact on the prognosis of penile cancer patients (6–11).

Regarding guideline-adherent ILND, Mistretta and colleagues demonstrated a 75% reduction in cancer-specific mortality and 58% in N1–3 stages (5). Furthermore, in a retrospective study involving 425 patients from 12 European and American centers, Cindolo et al. showed a 41% reduction in overall mortality and 49% when guidelines on primary tumor and lymph node management were strictly followed (12).

There has been an urge to centralize treatment of penile cancer in certified cancer centers despite the largely inconsistent results (13, 14). Kilsdonk et al. reported that centralized treatment in Great Britain since 2002 has resulted in a much higher uptake of organ-preserving surgery but not in an improvement of 1- and 5-year survival rates (15). Other studies have shown a favorable influence of treatment centralization on overall survival, though these effects are largely attributed to an adequate use of ILND and indication-specific perioperative chemotherapy (14, 16–18).

Reaching an established minimum caseload for specific tumor entities is an important criterion for certified cancer centers. To the best of our knowledge, no reliable data are available to date on the impact of caseload and treatment setting (academic vs. non-academic) on penile cancer guideline adherence. The purpose of this study was to examine the impact of hospitals’ annual caseload of penile cancer patients on adherence to key clinical aspects of current guideline recommendations. A questionnaire-based compilation of fictitious treatment decisions was distributed among urological chief physicians and senior staff members to determine a potential minimum caseload in specialized penile cancer centers.

## Materials and Methods

### Study Design, Participants, and Endpoints

The E-PROPS working group (European PROspective Penile Cancer Study) intends to collate the therapeutic procedures in penile cancer patients using three sequential modules. Module 1 involves data collection/evaluation of a questionnaire addressed to 681 urologists from 45 hospitals in Germany (n = 34), Austria (n = 8), Switzerland (n = 2), and Italy/South Tyrol (n = 1) in 2018. It contained 14 questions evaluating the position of respondents in their respective hospitals, their responsibility in treatment decisions, and their theoretical knowledge on surgery on primary tumors, inguinal/pelvic lymph node dissection, and perioperative chemotherapy in penile cancer patients. The following parameters of participating centers were additionally recorded: level of care (university hospital, maximum care hospital, specialized hospital, and primary care hospital), responsibility for penile cancer chemotherapy at the hospital (urology only, oncology only, or both), number of beds and staff, and the number of penile cancer patients treated in 2017.

The survey was established and analyzed in accordance with the STROBE criteria (19–22) and granted approval by the Institutional Review Board of the University of Regensburg.

Of 557 evaluable questionnaires, only those completed by chief physicians and senior staff members (n = 206) were selected for analysis, assuming that this occupational group is largely responsible for all treatment decisions in penile cancer patients. Specifically, this involved decisions in six case scenarios (CS): local treatment of primary tumor stages pTis (CS1) and pT1b (CS2), indication for inguinal (CS3) and pelvic (CS4) lymphadenectomy, and neoadjuvant (CS5) and adjuvant (CS6) chemotherapy.

The endpoint in all statistical tests was a guideline-adherent recommendation for the prespecified CS, whereby the accuracy of the answers was assessed according to European Association of Urology (EAU) guidelines on penile cancer applicable at the time (23).

The real annual caseload of penile cancer patients treated in all participating institutions was reviewed in unadjusted and multivariate analyses to assess the potential influence on guideline adherence. The inclusion of penile cancer caseload reported per center for 2017 was continuous and dichotomized into 1–5 vs. >5, 1–7 vs. >7, 1–8 vs. >8, and 1–9 vs. >9 patients. Dichotomization was based on the number of cases reported and introduced to potentially obtain an idea of a threshold for an annual caseload.

### Statistical Analysis

Metric variables are presented as medians and interquartile ranges (IQRs). The relationship between metric and categorical variables was investigated using Spearman’s rank correlation, between categorical variables using the chi^2^ test. The effect size of significant results is indicated by the rank correlation coefficient Rho, using the chi^2^ test with Phi. In both cases, a value of 0.1 corresponds to a weak effect, 0.3 to a medium effect, and ≥0.5 to a strong effect (24).

The independent influence of the annual caseload on guideline adherence was analyzed using multivariate binary logistic regression models with stepwise backward variable selection, whereby the following independent variables were included into the regression models: level of care (university hospital yes/no), responsibility for chemotherapy (oncology alone vs. urology alone or in cooperation with oncology), number of staff and beds in the hospital (continuously coded), and providing the following relevant treatment options regarding penile cancer in the hospital (yes/no): radiotherapy, organ preservation, laser therapy, and annual penile cancer caseload of the hospital (continuously coded, as well as according to the abovementioned dichotomies).

A probability of error of <5% was accepted as a significant result in all tests (*p* < 0.05). All statistical analyses were performed using SPSS^®^V26 (IBM, Armonk, USA).

## Results

### Descriptive Results

The overall response rate was 81.8%, with 557/681 questionnaires answered. In the study group considered here (n = 206), four (1.9%), 15 (7.3%), 40 (19%), and 147 (71%) of completed questionnaires came from hospitals in Italy/South Tyrol, Switzerland, Austria, and Germany, respectively. Answers from university (n = 99; 48%) and non-university hospitals (n = 107; 52%) were almost equally distributed. The participating clinics had 40 (IQR 33–53) beds with 16 (IQR 11–20) medical staff and treated six (IQR 3–9) penile cancer patients in 2017. Of the respondents, 182 (88%) performed penile cancer operations on their own. Radiation therapy, organ-preserving procedures, and laser therapy for penile cancer patients were available in 46%, 91%, and 82% of cases, respectively. Chemotherapy in penile cancer was performed by urology alone, urology and oncology together, or oncology alone in 35%, 21%, and 44% of cases, respectively.

University hospitals treated significantly more penile cancer patients per year than non-university hospitals, with seven (IQR 5–8) and five cases (IQR 2–9; *p* < 0.0001; Rho 0.269), respectively.

**Figure 1:**
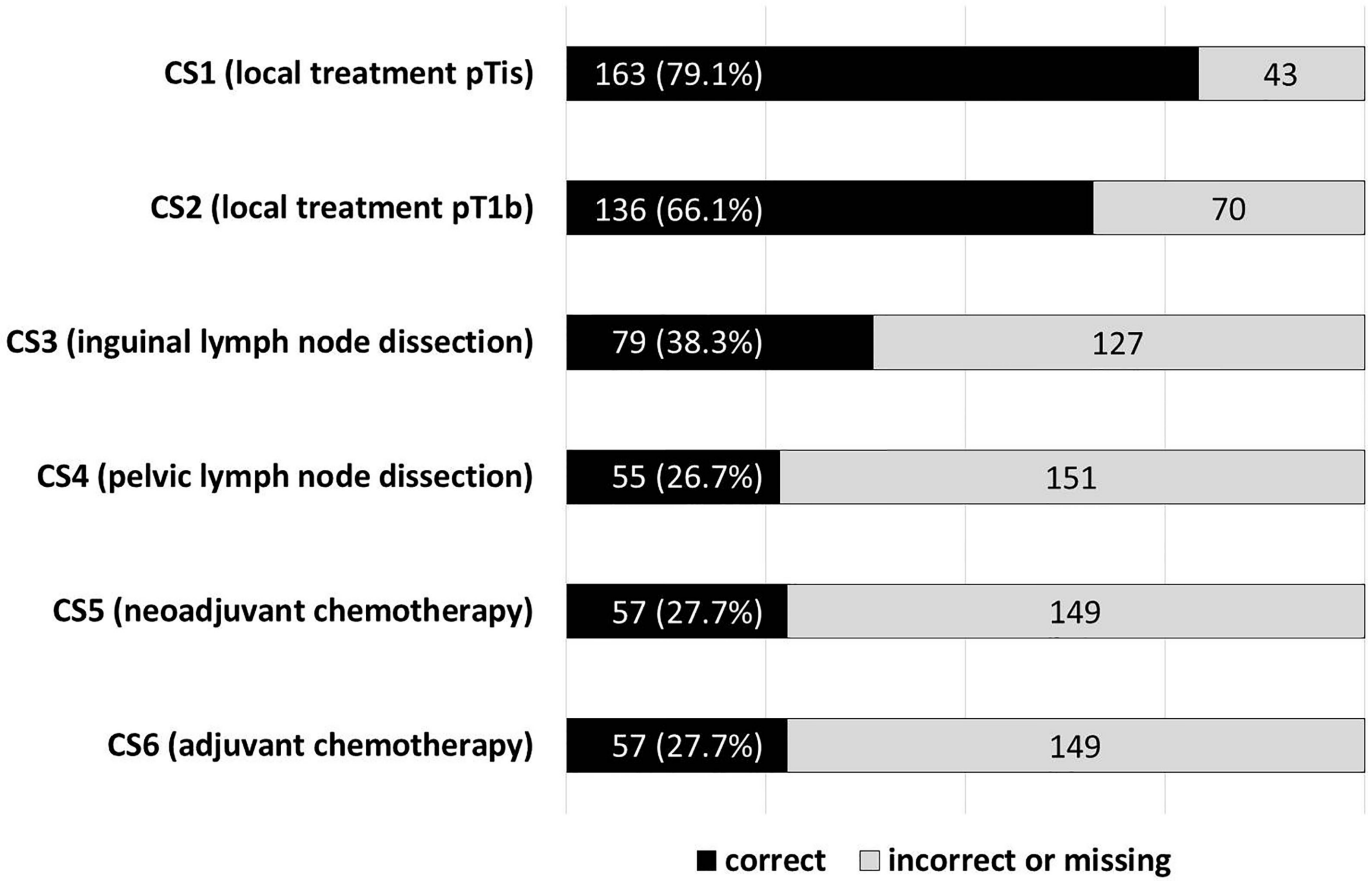
Proportion of treatment recommendations in line with the guidelines in terms of scenarios CS1–CS6. CSn, case scenario.

**Figure 1** shows an overview of the proportion of guideline-adherent recommendations with regard to scenarios CS1–CS6.

### Unadjusted Relationship Analysis Between Annual Penile Cancer Caseload and Guideline-Adherent Recommendation

With continuous inclusion of the caseload, recommended treatment only complies with guidelines for treatment of primary tumors in stages pTis (CS1) and pT1b (CS2), while the effect size (Spearman’s Rho) tends to be low (**Table 1**).

**Table 1:** Unadjusted relationship between number of cases and guideline-adherent treatment recommendation.

		Scenarios					
Inclusion		CS1	CS2	CS3	CS4	CS5	CS6
**Continuous**	*p*	0.001	0.016	n.s.	n.s.	n.s.	n.s.
	Rho	0.221	0.168				
**Dicho ≤5 vs. >5**	*p*	0.001	n.s.	n.s.	n.s.	n.s.	n.s.
	Phi	0.225					
**Dicho ≤7 vs. >7**	*p*	0.004	n.s.	n.s.	n.s.	n.s.	n.s.
	Phi	0.199					
**Dicho ≤8 vs. >8**	*p*	0.007	0.009	n.s.	n.s.	n.s.	n.s.
	Phi	0.188	0.181				
**Dicho ≤9 vs. >9**	*p*	0.025	n.s.	n.s.	n.s.	n.s.	n.s.
	Phi	0.156					

Significant results (p < 0.05) with indication of the effect size: Rho = effect size rank-sum correlation; Phi = effect size chi^2^ test.

Inclusion, inclusion of case number; CSn, case scenario; n.s., test result not significant.

With dichotomous inclusion of the annual caseload, a dichotomization of ≤8 vs. >8 cases seems to discriminate best between the chance of guideline-adherent and non-guideline-adherent recommendations, at least with regard to the therapy of the primary tumor. A higher caseload had a significant effect on CS1 and CS2, although the effect size (Phi) of <0.2 must be equally considered as low (**Table 1**).

### Multivariate Relationship Analysis Between Annual Penile Cancer Caseload and Guideline-Adherent Recommendation

**Table 2** shows the results of the multivariate analysis. Regarding the different CS, most often, an independent positive influence of the annual penile cancer caseload on the probability of a guideline-adherent recommendation for the treatment of the primary tumor in the pTis-stage (CS1) was observed.

**Table 2:** Multivariate relationship between number of cases and guideline-adherent treatment recommendation.

		Scenarios					
Inclusion		CS1	CS2	CS3	CS4	CS5	CS6
**Continuous**	*p*	0.003	0.023	0.023	n.s.	n.s.	0.012
	OR	1.16	1.08	1.07			1.10
	95% CI	1.05–1.28	1.01–1.15	1.01–1.14			1.02–1.18
**Dicho ≤5 vs. >5**	*p*	<0.001	n.s.	n.s.	n.s.	n.s.	n.s.
	OR	3.80					
	95% CI	1.79–8.03					
**Dicho ≤7 vs. >7**	*p*	0.006	n.s.	n.s.	n.s.	n.s.	n.s.
	OR	3.21					
	95% CI	1.40–7.35					
**Dicho ≤8 vs. >8**	*p*	0.031	0.011	n.s.	n.s.	n.s.	n.s.
	OR	3.38	2.68				
	95% CI	1.12–10.2	1.25–5.74				
**Dicho ≤9 vs. >9**	*p*	n.s.	n.s.	0.018	n.s.	n.s.	n.s.
	OR			2.44			
	95% CI			1.17–5.10			

Significant results (p < 0.05) with odds ratio (OR) and 95% CI.

Inclusion, inclusion of case number; CSn, case scenario; n.s., test result not significant.

Essentially, for four out of the six endpoints (CS1–3 and CS6), a significant influence of the annual caseload continuously included into the models on guideline-adherent recommendation was noted.

The dichotomized inclusion of caseloads presented a rather inconsistent picture: only a dichotomization at eight was able to predict two out of six endpoints. Respondents from clinics with an annual caseload >8 (compared to ≤8) made guideline-adherent decisions for surgical treatment of primary tumor stages pTis (CS1) and pT1b (CS2) at respectively 3.4 times (*p* = 0.031) and 2.7 times (*p* = 0.011) more frequently.

Regarding the indications for pelvic lymphadenectomy and neoadjuvant chemotherapy, an independent influence of the annual caseload on the probability of a guideline-adherent recommendation was not observed, neither for its continuous nor for its dichotomized inclusion (**Table 3**).

**Table 3:** Summary of the regression models on the influence of the annual caseload and other predictor variables on the probability of guideline-adherent treatment recommendations in the queried case scenarios.

Variable		Case scenario					
		CS1	CS2	CS3	CS4	CS5	CS6
**Number of penile cancer patients treated in 2017**	Elimination	No	No	No	Step 5	Step 5	No
(cont.)	p	0 .003	0.023	0.023	n.a.	n.a.	0.012
	OR	1.16	1.08	1.07			1.10
	95% CI	1.05–1.28	1.01–1.15	1.01–1.14			1.02–1.18
**Academic centers**	Elimination	Step 7	Step 3	Step 5	Step 2	Step 2	Step 6
(vs. non-academic)	p	n.a.	n.a.	n.a.	n.a.	n.a.	n.a.
	OR						
	95% CI						
**In-house patient capacity per department**	Elimination	Step 6	Step 4	Step 3	Step 7	Step 4	Step 4
(cont.)	p	n.a.	n.a.	n.a.	n.a.	n.a.	n.a.
	OR						
	95% CI						
**Number of urologists in the department**	Elimination	Step 4	Step 8	Step 2	Step 6	Step 6	No
(cont.)	p	n.a.	n.a.	n.a.	n.a.	n.a.	0.055
	OR						0.95
	95% CI						0.90–1.01
**Urologists performing chemotherapy for penile cancer patients**	Elimination	No	Step 7	Step 6	Step 8	No	Step 2
(vs. urologists not performing chemotherapy)	p	0.103	n.a.	n.a.	n.a.	0.030	n.a.
	OR	0.55				2.06	
	95% CI	0.26–1.13				1.07–3.96	
**Surgical organ-preserving treatments in penile cancer patients are provided**	Elimination	Step 2	Step 2	No	Step 4	Step 7	Step 7
(vs. no)	p	n.a.	n.a.	0.016	n.a.	n.a.	n.a.
	OR			0.26			
	95% CI			0.09–0.78			
**Local laser therapies in penile cancer patients are provided**	Elimination	Step 5	Step 5	Step 4	Step 3	Step 3	Step 5
(vs. no)	p	n.a.	n.a.	n.a.	n.a.	n.a.	n.a.
	OR						
	95% CI						
**Local radiotherapies in penile cancer patients are provided**	Elimination	Step 3	Step 6	No	No	No	Step 3
(vs. no)	p	n.a.	n.a.	0.089	0.064	0.028	n.a.
	OR			0.57	1.80	0.49	
	95% CI			0.30–1.09	0.97–3.37	0.25–0.92	

Green highlight = significant in the last step. Yellow highlight = no elimination, but insignificant in the last step. Orange highlight = elimination before the last step.

CSn, case scenario; OR, odds ratio; n.a., not applicable.

### Unadjusted and Multivariate Relationship Analyses Between the Level of Care in the Hospital and Guideline-Adherent Treatment Recommendations

In all CS, the level of care (academic vs. non-academic) did not significantly influence the probability of a guideline-adherent treatment recommendation, neither in an unadjusted model nor in a multivariate model (**Table 3**).

## Discussion

Satisfactory functional and cosmetic results of primary tumor surgery, as well as lymph node management, are considered essential cornerstones for the quality of surgical treatment in penile cancer.

This study was designed to give an indication of the minimum annual case numbers of specialized penile cancer centers based on international survey results from hospitals in Germany, Austria, Switzerland, and Italy/South Tyrol by using treatment recommendations of clinical decision makers.The qualification of the respondents is shown exemplarily in the high proportion of surgeons (88%) who performed penile cancer procedures independently. In a comprehensive evaluation of 409 procedures for penile cancer (USA 1998–2013) by Matulewicz et al., this only applied to 4.1% (346/8,545) of urologists included (25).

Despite this selection bias, the descriptive results (**Figure 1**) clearly show that four out of five respondents gave a guideline-adherent recommendation for the treatment of a pTis primary tumor, while in the case of a pT1b tumor, it was two out of three. In view of possibly considerable psychosocial implications of primary tumor treatment in penile cancer, this represents a worrisome finding (26). Although organ-sparing surgery carries an inherent increased risk of recurrence, they are not *per se* associated with compromised overall survival rates, thus providing a strong rationale for complying with guidelines to perform organ preservation when feasible (27).

Kirrander et al. showed a 5-year survival rate of 82% (95% CI 78%–85%) for penile cancer irrespective of tumor stage, which decreased to 46% (95% CI 36%–56%), particularly with the extent of nodal metastases. Nevertheless, guideline adherence recorded in this Swedish registry study on lymph node staging was only 50% in clinically normal inguinal lymph nodes in stages ≥pT1G2 (4). For neoadjuvant chemotherapy of patients with stage cN2-3 lymph node metastases, response rates of 43% (complete remission in 14%) were reported, with sustained remission in the adjuvant setting in a pN2-3 stage reported in 53% (median follow-up 42.6 months) (28–30). Particularly with regard to the prognosis-determining management of inguinal lymph nodes (including perioperative chemotherapy), recommendations that were not in adherence with guidelines predominated in our study. Based on these results, we believe that it is highly unlikely that a multimodal approach with adequate inclusion of neoadjuvant and/or adjuvant chemotherapy would be recommended in a N2-3 stage setting, where less than 30% of respondents actually gave a guideline-adherent treatment recommendation.

Based on the unadjusted and multivariate analyses of the correlation between the annual penile cancer caseload in a hospital and the likelihood of guideline-adherent recommendation, it was not possible to gain a reliable picture on a specific minimum number of cases. Although a statistically significant influence of caseload was shown in four out of six CS, with continuous inclusion (odds ratios of 1.07–1.16), every additional penile cancer patient treated per center translates into a 7%–16% increased chance of a guideline-adherent recommendation. At least regarding primary tumor treatment (pTis and pT1b), a clear increase in guideline-adherent recommendations was shown, where the annual caseload was ≥8 penile cancer patients. Although university hospitals treated significantly more penile cancer patients, the degree of care (academic vs. non-academic) demonstrated no influence on guideline-adherent recommendations.

Naturally, the results of a survey study set out here are not without important limitations. Even in a population-based sample area of 4 million and an annual minimum of 25 penile cancer patients, Kilsdonk et al. were unable to demonstrate a reliable effect on overall survival (15). In our own study group, 84% of respondents treated a maximum of 10 penile cancer patients annually in their hospital; another 14% treated 11 to 20, and only 2% treated 25 patients. None of the participating centers treated more than 25 penile cancer patients. In this respect, the number of patients in our study (n = 45 European hospitals, including 19 university hospitals) is probably too low to reliably demonstrate that guideline-adherent treatment recommendations correlate with case numbers. However, these caseloads do reflect a real-life scenario in countries where there is no legally underpinned centralization of penile cancer patients.

From a methodological point of view, regression models basically contain the risk of overfitting. Taking into account the relationship between predictors and number of events, step-by-step procedures, in particular the backward elimination, are a feasible way of counteracting overfitting.

In addition, we considered fictitious CS where each respondent was given individual recommendations; we did not consider treatments that were actually carried out. In daily clinical routine, it can be assumed that corresponding decisions are made by a number of qualified specialists or in an interdisciplinary tumor panel. Bearing this in mind, it might be further assumed that the proportion of guideline-adherent treatment decisions might be higher in reality.

Although it is hypothesized that the correlation between annual caseload, guideline-adherent treatment decisions, and functional as well as oncological outcomes might be confirmed in the case of penile cancer, solid and robust evidence underpinning the guidelines is somewhat limited (3, 14). The introduction of the EAU guidelines on penile cancer rightly points this out: “It must be emphasised that clinical guidelines present the best evidence available to the experts but following guideline recommendations will not necessarily result in the best outcome. Guidelines can never replace clinical expertise when making treatment decisions for individual patients, but rather help to focus decisions - also taking personal values and preferences/individual circumstances of patients into account. Guidelines are not mandates and do not purport to be a legal standard of care” (23).

## Conclusions

With a median annual caseload of six penile cancer patients per hospital, an indication for a significant correlation between the number of cases and guideline-adherent treatment recommendations could be hypothesized. However, in significantly larger study groups, no clear and significant effect of treatment centralization on penile cancer patients’ overall survival could be demonstrated, even in hospitals with a minimal annual caseload of 25.

Thus, the results of our study call for a stronger centralization of diagnosis and treatment strategies regarding penile cancer. This goal of course must not be compromised by possibly higher costs of travelling to high-volume centers, nor by personal, institutional, or even material interests.

## Data Availability Statement

The raw data supporting the conclusions of this article will be made available by the authors, without undue reservation.

## Ethics Statement

The studies involving human participants were reviewed and approved by Institutional Review Board of the University of Regensburg. Written informed consent for participation was not required for this study in accordance with the national legislation and the institutional requirements.

## Author Contributions

All authors participated in the conceptualization and writing of this paper, have seen and approved the final manuscript, and approved its submission.

## Conflict of Interest

The authors declare that the research was conducted in the absence of any commercial or financial relationships that could be construed as a potential conflict of interest.

## Publisher’s Note

All claims expressed in this article are solely those of the authors and do not necessarily represent those of their affiliated organizations, or those of the publisher, the editors and the reviewers. Any product that may be evaluated in this article, or claim that may be made by its manufacturer, is not guaranteed or endorsed by the publisher.
